# Childbirth rates in women with myeloproliferative neoplasms

**DOI:** 10.1038/s41375-024-02216-8

**Published:** 2024-03-09

**Authors:** Anna Ravn Landtblom, Therese M-L Andersson, Anna L. V. Johansson, Frida E. Lundberg, Jan Samuelsson, Magnus Björkholm, Malin Hultcrantz

**Affiliations:** 1 Department of Medicine, Solna, Karolinska Institutet, Stockholm, Sweden; 2 Department of Hematology, Karolinska University Hospital, Stockholm, Sweden; 3 Department of Medical Epidemiology and Biostatistics, Karolinska Institutet, Stockholm, Sweden; 4 Cancer Registry of Norway, Oslo, Norway; 5 Department of Hematology, University Hospital Linköping, Linköping, Sweden; 6 Department of Medicine, Myeloma Service, Memorial Sloan-Kettering Cancer Center, New York, NY USA

**Keywords:** Epidemiology, Cancer epidemiology

## Abstract

Myeloproliferative neoplasms (MPN) are associated with inferior pregnancy outcome, however, little is known about fertility and childbearing potential in women with MPN. In this study we aimed to describe reproductive patterns, as well as to quantify risk of miscarriage and stillbirth. Women aged 15–44 years with an MPN diagnosis 1973–2018, were identified in Swedish health care registers, and age-matched 1:4 to population controls. We identified 1141 women with MPN and 4564 controls. Women with MPN had a lower rate of childbirth (hazard ratio [HR] with 95% confidence interval was 0.78 (0.68–0.90)). Subgroup analysis showed that the rate was not significantly reduced in essential thrombocythemia, HR 1.02 (0.86–1.22) while the HR was 0.50 (0.33–0.76) in PV and 0.45 (0.28–0.74) in PMF. The risk of miscarriage was not significantly increased before MPN diagnosis, the HR during follow-up after diagnosis was 1.25 (0.89-1.76). Women with MPN were more likely to have had a previous stillbirth. Women with MPN had fewer children at diagnosis, and fewer children in total. In conclusion, the childbirth rate was lower among women with MPN than controls, but not among women with essential thrombocythemia.

## Introduction

Myeloproliferative Neoplasms (MPN) are a group of chronic hematopoietic malignancies, consisting of Polycythemia Vera (PV), Essential Thrombocythemia (ET), Primary Myelofibrosis (PMF), and MPN Unclassifiable (MPN-U) [1]. MPNs are more common among middle-aged and elderly individuals, however, 10-20% of patients are women of childbearing age at diagnosis [2, 3]. Pregnancy in MPN is complex, and increased risks of fetal loss, obstetric complications, and thrombohemorrhagic events have been reported [2, 4, 5]. Earlier studies have reported live birth rates around 70% in ET and 65% in PV, while in MPN-U and PMF there were too few pregnancies to estimate the live birth rate [2, 6]. We have previously performed a population-based study of all pregnancies in women with MPN in Sweden 1973–2017, and identified 342 pregnancies that had passed gestational week 22 (before 2008 week 28), and compared outcomes to matched control pregnancies. We found a significantly increased risk of preterm birth (12% vs 4%) but overall better fetal and maternal outcomes than previously reported [7]. This was in line with the only other prospective and population-based study in the field, by Alimam et al. [8]. Fertility issues and early miscarriages are of concern in MPN and requires further elucidation. To our knowledge there is no study that systematically describes reproductive patterns. The ability to bear children, and to have adequate information to make well-informed decisions regarding reproductive health is of major importance for quality of life in patients with chronic malignant diseases [9, 10]. In the current population-based study we compared women with MPN to age-matched controls, and describe childbirth patterns and quantify childbirth rates. Secondarily we also aimed to estimate fetal loss, miscarriage, stillbirth, and total number of children in women with MPN compared to population controls.

## Patients and methods

### Source population and central registers

Sweden is a country with 10.5 million residents where health care is publicly funded and maternal and pediatric care is free of charge. All residents have a personal identity number, that is used in all contacts with authorities and health care, and enables crosslinking between different registers [11]. The Swedish Cancer Register was founded in 1958, and since 1984 both pathologists and clinicians are obliged by law to report incident cancers, ensuring high data quality and completeness. However, as there may be a lower level of completeness for indolent cancers, we chose to also include diagnoses from the Patient Registers [12, 13]. The Inpatient Register was introduced regionally in 1964 and reached nation-wide coverage in 1987, and captures discharge diagnoses of all hospitalization periods [14]. The Outpatient Register has national coverage since 2001 and captures diagnosis of all visits to specialty clinics. The Medical Birth Register was started in 1973 and contains antenatal and delivery data on all pregnancies from week 22 (prior to 2008 from week 28) with a completeness of 97–99% [15]. These health care registers are held by the National Board of Health and Welfare. Statistics Sweden holds the Register of Total Population, with data on death and migration, and the Multi-Generation Register, which is a population register, linking all individuals that are born 1932 or later, and has resided in Sweden at any time point from 1961, to their parents [16].

### Study participants

Women in Multi-Generation Register was the source population for this study. From this population, all women with a diagnosis of MPN between 1973 and 2018, and aged 15–44 years at time of diagnosis were selected. The MPN diagnosis could be identified through the Swedish Cancer Register, Inpatient Register, or Outpatient Register. If the diagnosis was identified from the Outpatient Register, two separate occasions with a diagnosis of MPN were required. For each woman with MPN, four controls matched by age were identified from Multi-Generation Register. The date of the MPN diagnosis was defined as the matching date. The controls were required to be alive and residing in Sweden at the matching date. MPN patients and controls were excluded if they had received a diagnosis of another hematologic malignancy at the matching date. Censoring was at death, emigration, other hematologic malignancy, turning 45 years of age, or end of study, December 31st 2018.

### Outcomes

The main outcome was time to first live childbirth after MPN diagnosis. Information on live births were obtained from the Multi-Generation Register. Secondary outcomes included miscarriage and stillbirth before and after MPN diagnosis. Miscarriage included spontaneous and missed abortion, and was identified by ICD10 (O03, O02.1) from Inpatient and Outpatient Register and was available from 2001 and after. Stillbirth was defined as fetal loss after gestational week 22, prior to 2008 from week 28, and was obtained from the Medical Birth Register.

Baseline data regarding previous parity, history of miscarriages and stillbirth at matching date were analyzed in women with MPN and controls. Previous miscarriage, captured from 2001 and after, recent miscarriage within two years prior to diagnosis, and repeated miscarriage, three or more, were analyzed among women with matching date of 2003 or later. Total number of children, both at matching date and for women turning 45 years of age during follow-up was also assessed.

### Statistical methods

**Fig. 1:**
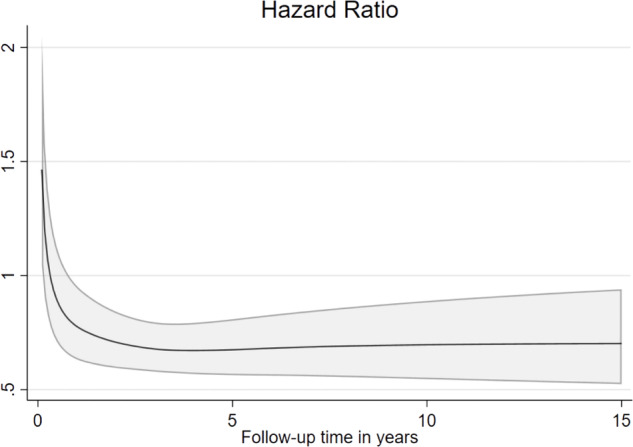
Hazard Ratio with 95% confidence intervals of childbirth in MPN patients versus controls in relation to follow-up time in years. MPN Myeloproliferative neoplasms.

**Fig. 2:**
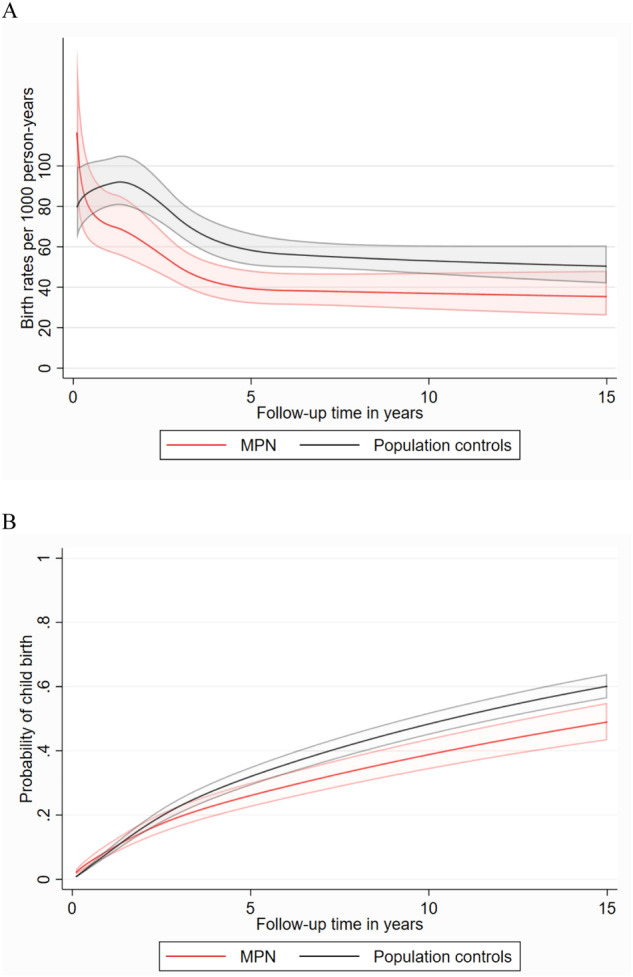
Childbirth rates and cumulative incidences of childbirth. Childbirth rates (**A**) and cumulative incidences (**B**) with 95% confidence intervals, estimated from non-proportional flexible parametric models, for patients and population controls aged 26–35 at diagnosis and diagnosed during the years 1989–2003. In cumulative incidence, competing risks were not accounted for. MPN Myeloproliferative neoplasms.

Flexible parametric models were used to estimate childbirth rates in MPN patients and controls and hazard ratios (HRs) with 95% confidence intervals (CIs), with time since MPN diagnosis or matching as the timescale. The analyses were adjusted for age at diagnosis/matching date (15-25, 26-35, 36–44 years), and calendar year of diagnosis/matching date (1973–1988, 1989–2003, 2004–2018). Proportional hazards were assumed for estimating the overall HRs, while models allowing for non-proportional hazards where also fitted to demonstrate how the rates and HR changes over time (Figs. 1–2). Cumulative incidence (1 minus the survival) was calculated using a non-proportional flexible parametric model, where competing risks were not considered.

For the main outcome, childbirth rate, separate analyses were done by age category, (15–25, 26–35 and 36–44), and MPN subtype. In order to exclude participants that were pregnant at diagnosis, and to reduce the potential risk of detection bias related to pregnancy and miscarriage, we performed a sensitivity analysis of both childbirth and miscarriage. Follow-up was then started at 9 months after matching date, applying the same censoring criteria and adjustments as in the main analysis. In an additional sensitivity analysis, we performed separate analysis by source of MPN diagnosis.

When comparing proportions in baseline data, Fisher exact test was used, and for means Student’s *t*-test, *p* < 0.05 was considered statistically significant. All statistical analyses were performed using Stata, version 16 (Stata Corp, Texas, United States), and SAS software, version 9.4 (SAS Institute Inc. Cary, NC United States).

Ethical approval was granted from Ethical Review Board (approval number 2020-05539), informed consent was waived since there was no contact with the study participants.

## Results

### Study population characteristics at diagnosis

The study population consisted of 1141 women with MPN and 4564 controls. Median age at diagnosis/matching date was 36 years, interquartile range 29–40 years. Of the women with MPN, 268 (23.5%) had PV, 620 (54.3%) ET, 120 (10.5%) PMF, and 133 (11.7%) MPN-U, Table 1. Among women with MPN, 61.0% had given birth to one child or more at the time of the MPN diagnosis, compared to 67.3% in controls (<0.001).

**Table 1:** Study population characteristics at MPN diagnosis/matching date.

	MPN Total 1141 *n* (%)	Controls Total 4564 *n* (%)
Age: 15–25	174 (15.2)	696 (15.2)
26–35	357 (31.3)	1428 (31.3)
36–44	610 (53.5)	2440 (53.5)
Subtype: PV	268 (23.5)	NA
ET	620 (54.3)	NA
PMF	120 (10.5)	NA
MPN-U	133 (11.7)	NA
Calendar year: 1973–1988	158 (13.8)	632 (13.8)
1989–2003	383 (33.6)	1532 (33.6)
2004–2018	600 (52.6)	2400 (52.6)

*MPN* myeloproliferative neoplasm, *PV* polycythemia vera, *PMF* primary myelofibrosis, *MPN-U* MPN Unclassifiable.

### Childbirth rate

The childbirth rate was reduced by 22% in MPN women compared to controls, HR 0.78 with 95% CI 0.68–0.90. The total follow-up time was 27,612 person-years in the main analysis, mean follow-up time was 4.8 years, range 0–28.1 years (3.4 for MPN and 3.8 for controls). Total number of events, i.e. first live childbirth after MPN diagnosis was 221 among MPN patients and 1131 among controls. There was no statistical evidence of an interaction between previous parity and childbirth during follow-up, with similar HR for nulliparous women was 0.79 (0.66–0.95) and in parous 0.74 (0.58–0.93), (*p* for interaction = 0.619). There was evidence of non-proportional hazards, which was mainly driven by a higher birthrate among MPN women shortly after diagnosis, since there were MPN women that were pregnant when they were diagnosed with MPN. Otherwise, the HR was fairly stable in relation to time after MPN, Fig. 1. The childbirth rates in MPN patients and controls are shown in Fig. 2A. The cumulative incidence illustrates that a lower proportion of women with MPN than controls give birth after diagnosis/matching date, Fig. 2B.

### Childbirth rate in relation MPN subtype and to age

In this cohort of MPN patients of childbearing age, ET was the most common MPN subtype and interestingly, in ET patients, childbirth rates were similar to controls, HR 1.02 (0.86–1.22). The childbirth rates were significantly reduced in the other MPN subtypes, PV 0.50 (0.33–0.76) and PMF 0.45 (0.28–0.74). Birthrates were significantly reduced in women aged 15–25, but not evident in the other age categories, Table 2.

**Table 2:** Hazard Ratio of live birth per MPN subtype and age at diagnosis only first live birth during follow-up is included.

	Events in MPN	Events in controls	Hazard Ratio	95% Confidence interval
All	221	1131	0.78	0.68–0.90
Age: 15–25	69	424	0.64	0.49–0.82
26–35	128	612	0.83	0.69–1.01
36–44	24	95	1.06	0.68–1.65
Subtype: PV	26	177	0.50	0.33–0.76
ET	156	676	1.02	0.86–1.22
PMF	18	150	0.45	0.28–0.74
MPN-U	21	128	0.52	0.33–0.82

*MPN* myeloproliferative neoplasm, *PV* polycythemia vera, *ET* essential thrombocythemia, *PMF* primary myelofibrosis, *MPN-U* MPN unclassifiable.

### Miscarriage and stillbirth

There were 636 patients with MPN and 2544 controls with matching date 2003 or later, available for analysis of history of miscarriage. At diagnosis, the proportion of women that had experienced miscarriage (≥1, after 2001), recent miscarriage (within 2 years prior to diagnosis), or repeated miscarriages (≥3) was similar between MPN patients and controls, Table 3.

**Table 3:** Previous parity, miscarriage, and stillbirth at diagnosis.

	MPN Total 1 141 *n* (%)	Controls Total 4 564 *n* (%)	*p*-value
Mean number of children	1.29	1.42	**0.001**
Parous at diagnosis	696 (61.0)	3 073 (67.3)	**<0.001**
Miscarriage^a^	79 (12.4)	318 (12.5)	1.000
Recent miscarriage (within 2 years)^a^	16 (2.5)	59 (2.3)	0.770
Repeated miscarriage (≥3)^a^	14 (2.2)	54 (2.1)	0.879
Stillbirth	11 (1)	16 (0.4)	**0.013**

*P* < 0.05 is considered significant (bold).

*MPN* myeloproliferative, *PV* polycythemia vera, *PMF* primary myelofibrosis, *MPN-U* MPN Unclassifiable.

^a^Miscarriage data is available from 2001, and was analyzed in women with diagnosis/matching from 2003 and onwards, total number is 636 MPN patients and 2544 controls.

In the analysis of miscarriage during follow-up, from 2001 and after, there were 705 women with MPN and 2820 controls. The mean follow-up time was 5.0 years and the total observation time was 17,475 person-years. There were 43 events of a miscarriage among MPN patients during follow-up, and 140 among controls. HR of miscarriage was 1.25 (0.89–1.76). In the sensitivity analysis, starting follow-up 9 months after matching date, HR of miscarriage was 1.06 (0.72–1.55).

One percent of women with MPN (*n* = 11) had experienced a previous stillbirth at diagnosis compared to 0.4% (*n* = 16) of controls, a statistically significant difference (*p* = 0.013).

There were too few events of stillbirth during follow-up for statistical analysis, one woman with MPN and six controls experienced stillbirth during follow-up.

### Total number of children

At diagnosis, women with MPN had an average of 1.29 children, compared to 1.43 in controls, *p* = 0.001. In the 652 women with MPN and 2904 controls that turned 45 years of age during the study follow-up, 82.2% of women with MPN had ever given birth to a child and 87.5% of controls. Mean total number of children, both before and after diagnosis, was 1.82 in MPN and 2.01 in controls.

### Sensitivity analysis

In the sensitivity analysis where follow-up was started 9 months after diagnosis, 953 MPN patients and 4022 controls were included and followed for a total of 23,532 person-years. The childbirth rate in women with MPN was reduced similarly to the main analysis, (HR 0.72, CI 0.61–0.89). In the sensitivity analysis based on source of inclusion, the HR of birthrate was similarly reduced in Cancer Register and Inpatient Register, but not significantly reduced in patients identified from Outpatient Register.

### Reasons for censoring

To investigate potential differences between MPN patients and controls, we explored reasons for censoring during the study follow-up. Most common reasons for exiting the study was turning 45 years of age or end of study, however censoring due to death occurred in 57 (5.0%) MPN patients, compared to 19 (0.4%) among controls. Causes of death in MPN was registered as acute myeloid leukemia or myelodysplastic syndrome (MDS) in 8 women (14%), vascular event in 13 (23%), other cancer in 14 (25%), infection in 7 (13%), and other causes in 15 (26%). Other hematologic malignancy was the reason for censoring in 29 (2.5%) MPN patients and in 3 (0.1%) controls.

## Discussion

In this large population-based study, we found a lower childbirth rate in women with MPN compared to controls. Overall, women with MPN had a lower number of children compared to the control population. The number of stillbirths was increased prior to MPN diagnosis, after which there were too few stillbirths for an adequate analysis. There was no significant difference in the risk of miscarriage before or after MPN diagnosis in relation to the controls. In subgroup analysis HR of childbirth was not reduced in ET, whilst it was significantly reduced in PV and PMF. Childbirth rates tended to be more affected in women aged 15–25 years at diagnosis, compared to their age-matched counterpart. To our knowledge, this is the first population-based study addressing childbirth rate in women with MPN.

Fertility and childbearing in women with cancer and chronic disease are important for quality of life [9, 10, 17]. There is a general trend in many developed societies of delaying childbirth in life; mean maternal age at childbirth in Sweden was 26.0 in 1973, and has gradually increased to 30.5 years in 2018, [18] suggesting that a larger group of women will receive a diagnosis of MPN prior to having completed their family plans.

In the subgroup analysis, the low childbirth rate was mainly driven by reduced HRs of childbirth in women with PV and PMF while the rate was not significantly reduced in women with ET. This is interesting as the majority of women of fertile age with MPN have ET. In general, PV and PMF have a more pronounced impact on the life expectancy as well as the risk of vascular complications [19–21] and the underlying disease mechanisms may similarly impact fertility and pregnancy related complications.

Younger MPN patients may be treated with observation alone, aspirin, phlebotomy, pegylated interferon-α or anagrelide, according to Swedish guidelines, in line with international recommendations [22]. Other agents in younger patients are uncommon [23]. Pregnancy management in MPN includes aspirin during pregnancy and low molecular weight heparin during the postpartum period. If the pregnancy is considered high risk, pegylated interferon-α and low molecular weight heparin is recommended throughout the pregnancy [6, 22, 24]. Aspirin and interferon are considered safe in pregnancy, and can improve pregnancy outcomes in MPN [4, 25, 26]. Interferon-α does not pass the placental barrier [27]. Whether fecundity and fertility are affected by interferon-α in widely used doses in MPN is currently unknown. Aspirin is well-studied in the context of pregnancy in patients with antiphospholipid syndrome and preeclampsia, where it increases the chances of a successful pregnancy outcome, and also seems to increase fecundity when used around the time of implantation [28, 29]. Anagrelide and hydroxyurea are generally not advised to use during pregnancy [24]. In conclusion, common treatment options are unlikely to have a major negative impact on childbirth rates and fertility in MPN patients.

Previous reports on pregnancy in MPN patients have largely focused on maternal and fetal outcomes. These studies have not addressed the question of overall patterns of childbearing and number of children in women with MPN. One questions that is difficult to address through register-based data is if having an MPN diagnosis affects the women’s attitudes and reproductive choices, thus if women choose not to have children due the MPN diagnosis. There is also a question whether there could be an underlying MPN-related biological effect on fertility and the possibility of conceiving a child, or maintaining pregnancy as has been reported in other diseases with thrombotic implications such as anti-phospholipid syndromes [30]. Miscarriages have previously been reported to occur in 20–30% of pregnancies in ET, and 12–30% in PV [2, 31–34]. Miscarriage is common in the general population, the proportion of recognized pregnancies ending with miscarriage is around 8–15% and numbers as high as 30% have been reported. In the current study, we did observe a trend towards a higher rate of miscarriages in women with MPN, however not significant.

Strengths of this study is the size, with a large number of women with MPN in fertile age included and the use of matched controls for comparison. The population-based selection of patients and the use of prospectively collected data from high quality registers reduces certain forms of bias. Limitations include the lack of detailed individual information on included subjects. We do not have information on why birthrates are lower, whether it is due to patient’s choice or reduced fertility. The initially higher childbirth rates in MPN may be due to reverse causation, that women who are pregnant or planning pregnancy have an increased health awareness that may lead to an earlier diagnosis of an MPN. Also, a lower proportion than expected of the women with MPN were found in the Cancer Register. An imbedded weakness in the analysis is that the rate of fetal loss is dependent on the rate of pregnancies. Theoretically, a lower birthrate in MPN patients in combination with a similar rate of miscarriages could constitute an excess risk of miscarriage. There is also a risk of underreporting of miscarriages, however we have no reason to believe that there are any major differences in medical attention seeking behaviors regarding miscarriages between MPN patients and controls.

To summarize, this large and population-based study is the first to assess childbearing patterns in women with MPN. Overall, we found that childbirth rates were significantly lower in women with MPN. However, the difference was not significant for women with ET. We found that childbirth in MPN is more common than previously anticipated, in particular during recent years. Thus, optimizing MPN management before and during pregnancy with the goal to improve birthrates and minimize the risk of complications is of highest importance and warrants continued international collaboration on treatment guidelines. Findings from this and our previous study on pregnancy outcomes provide important information on childbearing potential, fetal, and maternal outcomes in MPN. Taken together, the results enable informed conversations on family planning and prognosis of childbearing and pregnancy and convey a message that childbirth with low risks of complications is possible in the majority of patients with MPN.

## Data Availability

According to ethical permissions, data sharing by the authors to a third party is not allowed. The data is accessible by application to the Swedish National Board of Health and Welfare and Statistics Sweden.
